# Erasing traumatic memories: when context and social interests can outweigh personal autonomy

**DOI:** 10.1186/s13010-014-0021-6

**Published:** 2015-02-22

**Authors:** Andrea Lavazza

**Affiliations:** Centro universitario Internazionale, Via Garbasso 42, Arezzo, 52100 Italy

**Keywords:** Memory-erasing, Neuroethics, Autonomy, Social interest, Neuroscience

## Abstract

Neuroscientific research on the removal of unpleasant and traumatic memories is still at a very early stage, but is making rapid progress and has stirred a significant philosophical and neuroethical debate. Even if memory is considered to be a fundamental element of personal identity, in the context of memory-erasing the autonomy of decision-making seems prevailing. However, there seem to be situations where the overall context in which people might choose to intervene on their memories would lead to view those actions as counterproductive. In this article, I outline situations where the so-called composition effects can produce negative results for everyone involved, even if the individual decisions are not as such negative. In such situations medical treatments that usually everyone should be free to take, following the principle of autonomy, can make it so that the personal autonomy of the individuals in the group considered is damaged or even destroyed. In these specific cases, in which what is called the “conformity to context” prevails, the moral admissibility of procedures of memory-erasing is called into question and the principle of personal autonomy turns out to be subordinate to social interests benefitting every member of the group.

## Introduction

Neuroscientific research on the removal of unpleasant and traumatic memories is at a very early stage, although in recent years there has been significant progress in the understanding of the mechanisms of memory and its possible alterations [1]. The main purpose of those practices is to give relief to those who have experienced or witnessed negative events (accidents, assaults, natural disasters, terrorist attacks) which caused serious psychological consequences and, in severe cases, even led to post-traumatic stress disorder (PTSD). In this sense, unpleasant memories should be distinguished from traumatic memories. Unpleasant memories are those we’d rather not have, as they can jeopardize our serenity, but typically do not lead to any known pathologies. Traumatic memories, on the contrary, are memories that often lead to PTSD and other psychiatric disorders, memories whose negative emotional charge prevents one from leading a fully “normal” life.

In addition to forms of psychotherapy that use the mechanisms of memory extinction in the reconsolidation window [2], the drug currently most tested on humans is propranolol. It is a beta-blocker molecule that has the effect of mitigating the emotional burden of memories, if taken a few hours after the negative event. The semantic memory of the fact is not affected, while the physiological arousal associated with emotions is greatly reduced. The effectiveness of propranolol, however, has not been fully determined yet [3-6].

The recently investigated fact that long-term memory is susceptible to disruption after a consolidated mnestic trace is retrieved has led to try other innovative treatments of fear memory [7]. Specifically, every time a memory is retrieved a window of lability opens up and reconsolidation subsequently occurs [8]. When a protein synthesis inhibitor is given after retrieval, molecular and cellular mechanisms of reconsolidation are disrupted and long-term memories are significantly impaired on subsequent tests [9]. Although that technique has only been used in animal models, it may be feasible for use in humans. “Theoretically, patients could be brought into a clinical setting, presented with a stimulus that retrieves the fearful stimulus and given a drug, and the fear memory would be weakened” [7].

Other substances and other techniques (from the ablation of individual neurons to optogenetics) [10,11] have only been tested on animal models so far and experiments on humans still seem far away. Furthermore, it has also been argued, at the conceptual level, that it could be difficult (if not impossible) to erase memories that are woven into a strongly interconnected semantic network.

Still, the theoretical possibility of eliminating memories in humans has aroused a significant philosophical and neuroethical debate [12-15]. There seem to be two main contending paradigms: a “liberal” one and a “conservative” one [16]. In short, the former refers to the principle of individual autonomy in a perspective linked to J.S. Mill’s harm principle, while the latter is related to the idea of authenticity and nature as a given that one should accept and respect. Although the arguments presented by both sides are strong and convincing, it seems that the first has more persuasive resources in the context of contemporary philosophy. It also seems to better capture the widespread view of the general audience, given that it mirrors the prevailing individualistic trend.

It is worth recalling that the concept of autonomy “is generally understood to refer to the capacity to be one's own person, to live one's life according to reasons and motives that are taken as one's own and not as the product of manipulative or distorting external forces” [17]. Those who are autonomous can decide for themselves without interference from others or personal limitations; they can act according to a project of their own, designed without constrictions. Autonomy also concerns the freedom to decide what to believe in and the ability to weight the pros and cons of a given course of action.

Another fundamental element regards the awareness of the rules one establishes and follows; a central role in this is played by rational reflection, i.e. the ability to assess existing norms of conduct and traditions and to choose, with the necessary balance between rational and emotional aspects, which ones to follow and which to ignore. Another way of putting this is to say that being autonomous means having the critical ability to pay attention to the outcome of one’s deliberations and being able to be driven by one’s purposes. Being autonomous means being oneself, following one’s own considerations, desires, conditions and characteristics. It has to do with being what can be considered one’s “true self” – which, as such, becomes an indispensable value.

As I mentioned, the argument of autonomy, *prima facie*, has a greater plausibility and cogency than the objections to memory amendments proposed by the conservative front. This is probably due to the loss of strength of the substantive positions about human nature. In fact, there is no univocal consensus as to what the essence of personhood is and whether it is to be preserved, both because of the overcoming of boundaries in an evolutionary perspective, and because of the ideal of progress and continuous improvement, which implies that limits should be exceeded and borders pushed further.

The considerations in defence of the authenticity of the subject, challenged by the voluntary deletion of some memories (be they unpleasant or traumatic), rely on the idea of a core of the person that is objectively in becoming, but still remains an identifiable point of reference throughout the diachronic unfolding of existence (although that is not the only concept of authenticity available; for instance, according to Sartre being authentic consists in accepting that nothing and no one makes you choose what you choose). This core of the person consists of those distinctive features that maintain continuity in time and which one has to remain faithful to (within the limits of imperfection and self-deceit that are common to all human beings). Since a significant memory modification is likely to change personal identity, the conservative side, given its concern for preserving authenticity, cannot approve of such a modification.

However, the concept of authenticity seems to presuppose a centre of gravity, so to speak, endowed with a kind of firmness that modern cognitive sciences are trying to dismantle thanks to a growing body of experimental results. Our everyday understanding of ourselves as conscious, rational, and responsible agents may be mistaken. Conscious agency may be merely epiphenomenal [18,19], that is, the phenomenological expression of a machinery that prepares, decides and causes our behaviour [20]. But if the self and the will are not really what we believe they are, we are only left with a bundle of sensations – as Hume put it – and the unity of self turns out to be an illusion crafted by our consciousness (perhaps due to the advantage it historically provided). Therefore, it would make no sense to defend a particular memory in order to preserve a part of our identity, which would be changing and unstable anyway. That of the fragmentation of the self is surely a radical hypothesis, which maybe not even Hume would have endorsed, and other more nuanced options are surely available. Yet, it seems hard to deny that neuroscience – as well as the philosophers working along with it – puts into question the classical concept of authenticity.

### Autonomy and composition effects

The liberal perspective and the argument of autonomy – which defend one’s right to erase some of one’s traumatic memories – may be evaluated according to what I propose to call “the argument of conformity regarding memory-erasing”. This is the principle by which the overall status of the beliefs and attitudes of the individual can only be disconnected to a certain extent from the experiences of the individual himself and from the beliefs and attitudes of other members of the group, or from the wider environment. This disconnection, in particular as regards the manipulation of memory, tends to produce dysfunctional consequences, which in the case of memory removal end up undermining the very possibility to fully exercise one’s autonomy.

Something similar to the principle of “conformity” can be found in some considerations made by the President’s Council on Bioethics as regards the erasure of memories [12]. The new generation of psychoactive drugs can create a separation between (cerebral) happiness and the actions and experiences in the world. This could lead, in the most extreme scenario, to a form of solipsism following artfully reconstructed memories. As exemplified in the movie *Strange Days* by Kathryn Bigelow and James Cameron, where people become “dependent” on other people’s memories relived as if they were their own, the selective removal of negative experiences would make us desist from the effort to construct ourselves and our world according to the model of virtue and self-perfecting.

In the conservative perspective, this means not learning from negative experiences and running away from what is unpleasant instead of facing it. The result can be a state of passivity in the face of the objectively negative conditions of the society in which one lives. In a condition of alienation due to, say, an unjust law or an unfair distribution of wealth, one can go two ways. First, one can try to change the social situation so as to improve one’s condition. Second, one can remove one’s personal discomfort with a psychoactive drug or through (false) exciting experiences, such as in the film *Strange Days*, without even trying to change external conditions. Choosing this second strategy will not change the situation nor other people’s opportunity to change it. But if many people went for this option, changing objectively negative situations would become very difficult for those who wished to try.

Obviously, this is an extreme situation, as scientifically improbable as it is socially unrealistic. But there is a less science-fictional tendency, encouraged by psychoactive drugs, to consider feelings as ends in themselves and not as corresponding with actual realizations, which are only possible in lives immersed in a physical world and in interpersonal relationships. The use of psychoactive drugs could also lead to a gap between what we feel and what others feel, thus “counterfeiting” the perception of reality and one’s actions. In this sense, however, it seems that we are still linked to the idea of authenticity, namely the idea of a “naturalness” that should not be bypassed or removed. According to these ideas, only those who pass through all the trials of life can really be said to be “human.” This is a legitimate view, but it takes some elements for granted, while they should be the outcome of a strong moral argument.

The “conformity” principle proposed here implies that the personal autonomy of choice should act as a fundamental moral principle as regards the possibility to erase one’s memories. But it also implies that its concrete form should not be incompatible with the responsibility towards the conditions of the world in which one dwells. In fact, there are situations that we cannot change to our liking and are given as pre-conditions for the exercise of our autonomy. Enacting individual choices – which globally add up, thus amplifying their scope – can lead to changing those conditions in the world, nullifying (wholly or in part) the possibility of some autonomous choices and making the very exercise of autonomy dysfunctional^a^.

In other words, we will not enjoy the cerebral happiness offered by drugs for selective forgetfulness (or for other forms of enhancement) free of collateral preconditions, which depend largely on other people. A society dominated by the Hobbesian *homo homini lupus* rule could certainly stand as a realm of personal autonomy, but the state of continuous conflict would repeatedly put at risk our ability to self-determination, since violent interferences with our choices would be the norm. It therefore seems that the “rule” of autonomy should be implemented so that, even if everyone took it to the highest degree, it would still be possible for everyone to exercise it. Yet, this appears not to be always practicable. Consequently, it may be reasonable to put some limitations to the possible choices on the part of each subject, in addition to Mill’s minimal principle of not harming other people. I believe we should think in terms of real life, considering concrete situations that can actually occur, in order to realistically consider the conditions that are necessary for the exercise of autonomy itself.

This argument may seem to ascribe some value to personal continuity. In fact, personal continuity is functional to conformity in the sense in which I defined it. A person can make use of the principle of autonomy to act on her memory, altering in a way her continuity of existence, but doing so she can undermine the very conditions for her own autonomy. In this perspective, the continuity of existence, however, is not a value in itself, as the conservative stance legitimately claims. What matters is the conformity that prevents discrepancies between personal memory and external reality, with all the consequences this entails. It can therefore be said that the exercise of autonomy is based on an “instrumental” respect of a certain personal authenticity and continuity.

### Composition effects in practice

As a jurist, Adam Kolber [21,22] was among the first to show how the use of a substance that weakens the memory can create a conflict between *a)* the right of society to protect itself from criminals (prohibiting the destruction of the witness statements) and *b)* the right of people to control their own memories (i.e. their minds). If the victim of an aggression “deletes” the event from her memory or even just its emotional consequence, she may lose both the will and the ability to press charges. Society is thus also deprived of important information to make sure that the person responsible for the crime is prosecuted and other people are protected from other crimes. The physician prescribing propranolol (or another psychoactive drug) to a woman who was raped, with the result that the woman does not want or is unable to testify, would risk the charge of obstruction of justice.

The important point here, however, is the behaviour of the victim. She has a traumatic memory, one that most likely will create permanent damage. Does she have the right to cancel the memory, even if the criminal will thus remain free and unpunished? The principle by Mill here should be declined in a specific way. It does not seem, at first sight, that by manipulating her own mind to prevent a psychiatric disorder the person assaulted is doing damage to others. But not pressing charges (or not testifying) against a potentially serial rapist who attacked us is like not stopping a rapist who is running away after attacking someone else. If everybody adopted this behaviour, the safety of society as a whole would deteriorate severely. This is not only because many criminals would remain free and unpunished, but also because the growing awareness that many victims prefer to forget rather than pressing charges would increase the propensity to commit crimes. Many people who currently do not commit crimes for fear of the legal consequences might decide that it is worth the risk, given the low probability of getting caught.

It can be argued that, in this way, people forced to keep a traumatic memory are treated not as an end but as a means for the welfare of society. In fact, some people seem to have a more than justified claim to remove their traumatic memories – compared to merely unpleasant ones – because they have been victims of criminal acts and not, for example, of accidents due to their own carelessness (provided that this can make a difference). It can be argued, however, that each victim who refuses to report or testify, preferring to delete their traumatic memory, helps to make the environment even less secure, to their own damage. The risk of a new, even deadly attack will be more likely. If one is killed, of course, any opportunity to exercise one’s autonomy is reduced to zero: an unconstrained exercise of autonomy can thus undermine the basic conditions of autonomy itself^b^.

Speaking of using people as a means, consider another scenario. Holocaust survivors, had it been available at the time, would have had good reasons to take a psychoactive drug that could mitigate or erase the terrible memories of the death camps. An event so devastating destroyed forever the lives of many of those people, so much so that some of them have committed suicide. On the other hand, the preservation of the memory of the crimes perpetrated and the attempt to make future generations understand the Holocaust are generally considered the best tools for this kind of event not to happen again. Survivors devoting part of their lives to this task are not be mere instruments, but active agents to a noble end surely worthy of being pursued, which would compensate (at least partly) the price of not removing the trauma.

It could be argued that, because of the composition effects, a single witness of the Holocaust who chose to forget her experience for her own personal well being would be justified and would not be harming society. But if everyone chose to do so, the effect would be disastrous. Perhaps a pragmatic solution, which does not conflict with any of the principles under discussion, would be to allow the treatment to those who show the greatest signs of suffering. Or, since the list of survivors is known, one might ask an adequate number of survivors to “volunteer” to keep their memories in order to preserve the direct remembrance of extermination camps.

Negative composition effects arising from the combination of several individual choices are not rare, even in contexts other than memory-erasing. Such composition effects end up turning against the individuals’ very choices, so that a central coordination limiting individual autonomy, or even a prohibition of choice, may end up being better not only globally, but also for each individual decision-maker. The issue of gender-based selection of unborn children could offer an example here. Despite the argument in favour of the parents’ autonomy of choice (leaving aside the moral considerations about the suppression of embryos), on a broad scale the overall effect on society could lead to severe imbalances in the quantitative ratio between males and females – an unintended outcome emerging from the individual freedom of choice.

The prevalence of males or females could then lead to social imbalance (lack of wives or husbands, lack of workers with certain characteristics, gender tensions) that would be negative for many (even though not for everyone, as some could even benefit from it), outweighing the benefit of being able to chose to have a child of a given gender. Individual preferences, family welfare and the welfare of the new-born could also be nullified by the unpredictable situation following from such choices. For example, one may prefer a male because there are few in the proximal environment, and then find out that everyone else has had a son in the meantime. Furthermore, the advantage/disadvantage of being of a given gender can change with the new ratio in the younger generation and so on. Therefore, prohibiting or restricting gender selection can be rational and justified, even at the expense of people’s freedom of choice.

### The example of abused women

Back to memory-erasing, if many people freely decided to use a hypothetical drug that can eliminate guilt (erasing the memories of negative deeds done by the subject), we would risk having a much more inhospitable society (this would not concern violations of the law, but the instinctual benevolence that generally keeps us from mistreating our neighbours). If no one were willing to bear in mind the horror one witnessed, we would risk living in a world where atrocities are easily repeated, making it more difficult to exercise autonomy itself. In these cases, however, we are talking about too broad and general scenarios, in which counterexamples could be designed and proposed. Only in extreme scenarios would there be a composition effect strong enough to justify a restriction of individual choice about the partial deletion of one’s memories.

So, let’s take a “negative” example, one that is narrower and more compelling. Imagine the case of a strongly patriarchal society in which most women are subjected to domestic violence or sexual abuse. Generally speaking, victims suffer in silence out of fear and shame. On the one hand, men resort to physical abuse, on the other hand there is a social system that stigmatizes and marginalizes those who try to rebel. The situation is thus perpetuated, with women traumatized and deprived of any right. In such a context, sometimes the suffering or humiliation are so strong that some women think that nothing would be worse than staying in their condition, and therefore try to react. It can be said that some progress in the recognition of the dignity of discriminated groups has been achieved in this way, even though it’s not a universal solution, since the rebellion of individual women might make the general situation worse due to the violent reaction of the patriarchal society.

But let's say that in our supposed society a medical treatment becomes available (say, a pill) to erase the memory of all abuses, even the worst. Given the possibility of a risk-free relieving solution, the women in our thought experiment freely decide to take the drug. Thanks to the effect of the substance, the memory of the violence fades or vanishes and the trauma is absorbed, at least for some time. Thus, it is conceivable that the overall condition of women will get even worse. The sexist system will get even stronger, as the drug will make any attempt to protest even more unlikely.

Thus, even in the utilitarian perspective of the reduction of suffering, one could not speak of an actual achievement, given that the violence might become more frequent if males thought that women were not suffering that much, after all. In fact, the women taking psychoactive drugs would enjoy a temporary subjective relief to their pain, but their objective conditions would remain the same as before. Also, reducing the pain of the trauma means weakening pleasures that are just as great, such as emancipation and justice. One may then wonder whether to produce and distribute the psychoactive drugs in a male-dominated society would not lead to a negative outcome, further reducing the autonomy of women it started out to defend.

Of course, it can reasonably be argued that increasing the opportunity of choice is still a goal to be pursued. Secondly, one could argue that the autonomy exerted by subordinated women is a false autonomy. As to the first consideration, one could reply that having opportunities for choice is not necessarily a good thing *per se*, if the alternatives do not envisage an improvement – or at least, a potential one. A sufferer of chronic pain can be proposed to have his limbs cut as a decisive “treatment”; however, it is intuitive that the ability to chose this option does not necessarily improve his overall well-being. Such choice would only make sense within a perspective from which the elimination of pain is evaluated as preferable to the ability of living and moving independently (which is rather questionable).

As for autonomy with regard to one’s memory, women taking the psychoactive drugs can intuitively be considered capable of self-determination (although this may depend on one’s conception of autonomy: not all substantive accounts of autonomy may judge such persons to be fully autonomous). Now, the problem lies precisely in the exercise of rational self-determination at the individual level regarding the use of such a powerful tool, as in the case in question. In essence, each subject does what *prima facie* appears the best for herself (just as a hypothetical NGO secretly providing the drug supposedly does so for the good of everyone), while actually worsening the general situation. On the other hand, almost no one can be a decision-maker able to predict all the aggregate consequences of one’s decisions, even assuming the Kantian imperative to act according to a maxim which can become a universal law. Finally, even if we supposed that the case of abused women is not valid as a condition to exercise autonomy, the examples of the inhospitable and brutal society we have seen before would still be strengthened. In fact, if what matters are external conditions and social relations affecting the self-determination of those women, then using autonomy in a certain way would turn against autonomy itself, limiting it or even making it false.

It can also be argued that the example of abused women lends itself to different interpretations, that is, to three modes of argumentation. In particular, according to what we might call “Argument one”, if radical memory modification is used, each person’s capacity for self-control as well as their social freedom is immediately affected, while others aren’t. In this case, the typical liberal paradigm seems to be at work, with the possibility for everyone to choose how to act in their own best interest, without others being affected, according to Mill’s harm principle. Nevertheless, one could argue that abused women who erase their memories would somewhat remain trapped within an ideology of enslavement.

According to “Argument two”, the group of people who undergo radical memory modification have their capacity for self-control and their social freedom affected. Still, other social groups won’t see any reduction of social autonomy or diminution of civic liberty. In this case, there are composition effects to the sole detriment of the group of abused women who take psychoactive drugs for the removal of memories. The other members of society, however, still seem immune from the consequences of these choices.

Finally, “Argument three” goes as follows: if any sufficiently large number of people undergo radical memory modification in a society, not only will their own autonomy suffer, but everyone in the society will see their civic freedom affected in the long run. In this interpretation, even those who are not taking medication for the removal of the memories are damaged (unbeknownst to them, at least in a first stage). Why? It may be useful here to introduce a Hegelian perspective. The reason why patriarchal men would also suffer from less self-autonomy and less civic freedom is because they would stay trapped in the slavish ideology of that society, failing to enjoy the higher consciousness and ethical life coming from full reciprocity with women. This argument is more challenging and requires a degree of agreement on some values that are not instrumental, while some limitations of autonomy about taking psychoactive drugs can also descend from consequentialist considerations. Nevertheless, “Argument three” shows that interventions on memory may have important consequences at different levels, which would be wrong to underestimate and deserves further reflection.

### The conformity objection to memory-erasing

The conformity objection is also suitable for a recent example devised by Walter Glannon, [23] which is the following. A very emotional and anxious young scholar has a small *defaillance* during a conference that he considers very important to his career. The thought of that event continues to distress him and threatens to jeopardize his public activity. Thus he decides to take a drug that makes the emotional charge of memories fade so as to make the disturbing event disappear from his mind. According to Glannon, such decision is morally incontestable from all points of view. And yet, what led the young man to his first failure could be a structural weakness that, by taking the drug, he will not even begin to address, thus exposing himself to a number of other potential failures. And even though he will still be able to use the tranquilizing psychoactive drugs, his colleagues will not forget the signs of his inadequacy to the role. After being at peace and confident for a while the scholar might actually end up ruined. His “cerebral” world will eventually be challenged by external reality: that is, the fact that he is no longer valued as a scholar. Reality will knock at his door and, at that point, no medication will help – unless we want to posit a form of fact-denying solipsism.

It is not just that forgetting what others know (which is also an important factor to consider) is impracticable. It is that we have to deal with the basic conditions that constrain the exercise of our faculties. If one wants to live in society, or even in the slightest relationship with one’s fellow human beings, the choice not to deal with external “objective” situations becomes morally relevant because everyone (even implicitly, by the mere fact of being placed in a context of social interactions) takes on responsibilities that one cannot reject at will. Those who opted for oblivion, even in the full exercise of their autonomy, would be likely to become detached from reality in an ethically unacceptable way. This does not happen when deleting a single unpleasant memory (provided that one day it will be possible), but it does when entering an overall perspective about one’s memory. Every unwelcome memory can potentially become the target of cancellation, resulting in the construction of an altered biography that does not correspond to our real life. This detachment is what we have when our mental life and our perception of reality no longer match the perception that other people have. On the one hand this goes against the principle of authenticity and, on the other hand, it has important consequences both for the person who makes up her memory and for those with whom she interacts. And when the conformity between memory and reality is lost, the consequences are often dysfunctional and negative.

As I hope to have shown, it seems that only those who have never entered into binding relationships, such as a person who always lived alone in a forest, could be able to claim full sovereignty on her memory in all situations.

## Conclusion

Thus, the individual autonomy in memory-erasing seems to be subjected some limitations, which are not the result of an *a priori* moral argument, but follow from the consideration of the consequences of individual choices. Such limits derive from the constraints of social life, which entail that, in some cases, the very pre-conditions of autonomy could be put at risk by the massive use of techniques of memory-erasing. In the absence of a centralized coordination, which is in turn a form of restriction of autonomy, personal choice can backfire both on the individual who takes the choice and on the group to which she belongs. In this sense, the liberal perspective faces some moral limits that I suggest we call “principle of conformity regarding memory-erasing”^c^.

### Endnotes

^a^That can seem analogous to Charles Taylor’s argument for liberal communitarianism. In brief, Taylor [24] maintains that liberal political institutions are very valuable, but that they tend to produce actions that gradually chip away at them. He therefore proposes constraints on what is allowed that seem to go beyond what liberalism would permit, but that are actually justified by the need to protect liberal institutions themselves.

^b^Cognitive liberty theorists like Jan Christoph Bublitz and Reinhard Merkel [25] are against the interference of state and non-state entities with human cognition in general, and specifically with an individual’s mental “inner realm”. But while the first obligation they propose is to protect individuals from other people’s interference with their cognitive processes, the second obligation is to ensure that individuals have the freedom to modify their own consciousness and mental processes in any way they want, including direct cognitive intervention through memory-erasing drugs. This radical version of cognitive liberty is likely to dismiss the importance of social interests as ethical or political reasons for outweighing personal self-determination. The main argument of this paper is intended to be an objection to such view. I posit that although everyone should be free to take any drugs, following the principle of autonomy, there are *occasions* on which the individual choice to take *some* drugs can jeopardise the very bases of autonomy.

^c^Many thanks to two anonymous reviewers for their helpful comments and suggestions.
